# Impact of nurse-led advance care planning in a primary care setting

**DOI:** 10.1017/S1478951526102508

**Published:** 2026-05-12

**Authors:** Sumathi Devarajan, Rebecca Rdesinski, Shannon Sweeney, Eriko Onishi, Erin Gallivan, Harry Krulewitch, Seiko Izumi

**Affiliations:** 1 Department of Family Medicine, Oregon Health & Science University, Portland, OR, USA; 2 Department of Neurology, Oregon Health & Science University, Portland, OR, USA; 3 School of Nursing, Oregon Health & Science University, Portland, OR, USA

**Keywords:** Advance care planning (ACP), primary care, nurse coordinator, serious illness conversation (SIC) training

## Abstract

**Objectives:**

We aimed to evaluate patient and clinician experiences of advance care planning (ACP) conversations facilitated by an ACP nurse coordinator (ACP-NC).

**Methods:**

We used a sequential mixed-methods approach that included a patient survey, patient interviews, and clinician interviews at a family medicine clinic. Patients were invited into the study if they had reached a stable point in their ACP decision-making conversations with the ACP-NC or PCP (i.e. their goals and preferences were considered settled at the time) and were not imminently dying. Invitations to complete a survey were sent within 2 weeks of patients completing their ACP conversations. Patient interviewees were purposefully selected to vary on key attributes such as age, gender, and number of ACP conversations. An iterative sampling strategy was used for both patient and clinician interviews.

**Results:**

Ninety-three patients completed the survey, and 10 patients were interviewed. Six clinicians were interviewed. Sixty percent of patient respondents reported being very comfortable having ACP conversations. At the time of the survey, 79% had completed or revised their existing advance directive. The professional groups that patients most preferred to engage with, regarding ACP, were their primary care provider (87%), ACP-NC (70%), and palliative care specialist (61%). Patient interviews indicated that participants appreciated being referred to the ACP-NC within the clinic, describing her as a motivator and generous with her time, which facilitated thoughtful discussion of preferences and wishes. Clinician interviews identified limited time as a key barrier to conducting ACP conversations and saw a dedicated ACP-NC as a major benefit to allow patients to spend more time having these important conversations.

**Significance of results:**

Patients were open to engaging in ACP discussions. Integrating an ACP-NC within primary care teams may represent an acceptable and effective approach to promote the early initiation of ACP in primary care settings.

## Introduction

Understanding and sharing one’s personal values, goals, and preferences regarding future medical care at any age or health status is the cornerstone of advance care planning (ACP). The goal of ACP is to help ensure that people receive medical care that is consistent with their values, goals, and preferences (Sudore et al. 2017). These efforts help guide healthcare providers in partnering with patients to make informed, patient–centered decisions. Key components are the incorporation of patient autonomy and consent (Singer et al. 1996). For families experiencing the end-of-life of a family member, ACP is associated with less stress, anxiety, and depression (Detering et al. 2010). Engaging in ACP and having a living will is linked to a lower likelihood of dying in a hospital (Degenholtz et al. 2004; Bischoff et al. 2013). Additionally, written advance directives and end-of-life care discussions are associated with reduced use of aggressive medical interventions (Wright et al. 2008; Dalmau-Bueno et al. 2021).

Despite the recognized advantages of ACP and early initiation of ACP in ambulatory settings, primary care providers (PCPs) face barriers, such as inadequate time and specific training, to engage in ACP discussions (De Vleminck et al. 2013; Howard et al. 2018; Swiderski et al. 2022). Conversations are often postponed until the need for decision-making arises from changes in health conditions (Deptola and Riggs 2019; Ouchi et al. 2019). We created a model where a Registered Nurse in the primary care team (PCT) is designated as an ACP Nurse Coordinator (ACP-NC), with dedicated time to take the lead in ACP conversations and to whom patients could be referred (similar to a complex care management program in Rhode Island [Lally et al. 2020]). To promote understanding and education about ACP and the role of the ACP-NC among the PCT, we offered ACP training sessions adapted from the Serious Illness Care Program developed by Ariadne Labs (Ariadne Labs 2025) to PCT members, including physicians, advanced practice providers, registered nurses, social workers, and trainees. The goal of this study was to evaluate the impact of ACP-NC-led conversations from the perspectives of patients and clinicians in a primary care clinic.

## Methods

### Study design and setting

We used a sequential mixed-methods approach, including a patient survey, patient interviews, and clinician interviews to understand patient and clinician responses and experiences with ACP conversations. The study was conducted from June 2022 through December 2024 and took place at a family medicine clinic affiliated with an academic medical center in the Pacific Northwest.

### Intervention

A registered nurse (E.G.) in the PCT at the family medicine clinic was appointed as an ACP-NC with 0.60 FTE dedicated time to facilitate ACP conversations and follow-up with patients. She had 10 years of experience as a nurse care manager working in this clinic and had received the serious illness conversation (SIC) educator training from a coinvestigator (S.I.) who was a trained SIC educator. We also offered SIC trainings to clinicians in the clinic to promote a shared understanding of ACP and how to facilitate ACP with their patients. The SIC follows a guide that encompasses patient-tested language to pursue patients’ goals, values, and preferences (Ariadne Labs 2025). In collaboration with PCPs and administrators in the clinic, the research team created a workflow for PCPs and other clinicians to refer their patients to the ACP-NC.

In addition to the referrals from clinicians, the ACP-NC proactively identified and engaged patients who met specific criteria to initiate ACP conversations. Eligible patients included those who came in for an annual Medicare Wellness Exam (aged ≥65 years and enrolled in Medicare), who were indicated as high risk for serious illness in the Electronic Health Record (risk categories 3 or 4), who were recently discharged from the hospital, or who self-referred by asking their PCP, clinic nurse, or other clinic staff for information about ACP. The ACP-NC facilitated in-depth conversations with patients using the SIC model, and documented and communicated with the PCPs about the contents of the ACP conversations, and made suggestions for follow-up.

### Survey

Patients who had reached a stable point in their ACP decision-making conversations with the ACP-NC or PCP, and were not imminently dying, were invited to participate in the study. A “stable point” was defined as a stage in the ACP process at which the patient’s goals and preferences were considered settled at that time. That is, no immediate changes were being made or anticipated, no additional follow-up actions were needed, the patient was not awaiting discussions with family or loved ones, and no further ACP conversations with the care team or family were scheduled. It is acknowledged that patients’ goals and preferences may evolve over time in response to changes in health status, life events, or care needs, therefore subsequent follow-up ACP discussions may be required in the future. Invitations to the survey were sent within 1–2 weeks of concluding their ACP conversations. The invitations were extended either in person, by email through the patient portal, or by mail. For data collected electronically, we used an online survey platform (Qualtrics) and sent the survey link electronically. For data collected via paper, we sent a paper survey and included a self-addressed stamped return envelope. Additionally, 1 survey was administered by phone because the participant was unable to complete an electronic or written survey due to limited hand and arm mobility. The survey was developed by the research team and included 13 questions asking about patient perception, comfort level, and acceptability of ACP conversations (see appendix A). Descriptive statistics were computed, and a Wilcoxon–Mann–Whitney test was conducted to compare comfort levels between genders using SAS software (SAS Institute Inc., Cary, NC).

### Qualitative data collection

One experienced qualitative researcher (S.S.) conducted semi-structured interviews (see appendices B and C for guides). Ten interviews were completed with patients (*n* = 8) and caregivers (*n* = 2) who finalized their ACP conversations. Patient and caregiver interviewees were purposefully and iteratively selected (Dicicco-Bloom and Crabtree 2006) to vary on key attributes such as age, gender, and number of ACP conversations. Caregivers who had participated with patients were included to give a voice to participants who were deceased. Patients were recruited via email correspondence or by phone, and all interviews were conducted using video software or by telephone and were recorded with participants’ permission. We sought patients’ understanding of the steps of the ACP process, their comfort level, what was valuable, and what could have been improved in the ACP process. Patient interviews were conducted between November 2023 and March 2024 and were approximately 20–50 minutes in length. Interviews occurred on average 11 months after their ACP conversations were completed.

Additionally, 6 clinician interviews were completed to learn about how clinicians experienced ACP conversations and training. Clinicians from the participating family medicine clinic were recruited via email correspondence or by phone. Clinician interviews were conducted between March and May 2024 and lasted approximately 40–55 minutes.

### Qualitative data management and analysis

We analyzed interviews in real time so that emerging insights informed subsequent data collection, as needed. All interviews were professionally transcribed, de-identified, and reviewed for accuracy. Transcripts were entered into Atlas.ti (Version 9, Atlas.ti Scientific Software Development GmbH, Berlin, Germany) for data management and analysis. One researcher (S.S.) analyzed interviews using a 5-step inductive approach (Crabtree and Miller 2023), tagging text in the transcripts to code emerging themes. Findings were then discussed and refined based on group discussion. Each interview was examined, after which a cross-case analysis was conducted to make comparisons across interviewees’ experiences (Crabtree and Miller 2023).

This study was approved by Oregon Health & Science University Institutional Review Board (IRB #23954).

## Results

During the study period between June 2022 and December 2024, 31 clinicians in the study clinic and affiliated primary care clinics, and 35 family medicine residents participated in the SIC training offered in this project. The ACP-NC facilitated ACP conversations with 549 patients in this clinic.

### Survey results

Of the 549 patients who had ACP conversations with the ACP-NC, 156 patients met the study criteria and were invited to participate (i.e. patients who had reached a stable point in their ACP decision-making conversations with the ACP-NC or PCP, i.e. their goals and preferences were considered settled at the time, and were not imminently dying). Of the 156 patients invited into the study, 93 completed the survey, yielding a 59.6% response rate. Respondents were mainly older, non-Hispanic, white females, which is reflective of the clinic population (OHSU, Dept of Family Medicine). Demographic details are provided in Table 1.

**Table 1.** Demographics of survey participants (*n* = 93) Table 1 long description.

Age [mean (SD) (range)]	79.9 (7.7) (52–95)
	*n* (%)
Gender	
Female	64 (68.8)
Male	26 (28.0)
Unknown	3 (3.2)
Hispanic or Latino origin or descent	
No, not Hispanic or Latino	86 (92.5)
Prefer not to answer/missing	7 (7.5)
Race	
White	86 (92.5)
Asian	1 (1.1)
Black or African American	1 (1.1)
Prefer not to answer/missing	5 (5.4)
Highest level of education completed	
Some elementary, middle, or high school	2 (2.2)
High school/GED or some college or licensed certification	25 (27.2)
2- or 4-year college	32 (34.8)
Graduate school or higher	33 (35.9)
Survey type	
Email	56 (60.2)
Paper/phone	37 (39.8)
Total	93 (100.0)
Survey response rate	
Email	56/93 (60.2)
Paper/phone	37/63 (58.7)
Total	93/156 (59.6)

About a third (34.4%) of respondents reported having had an ACP conversation with only the ACP-NC, 39.8% had a conversation with both the ACP-NC and their PCP, and 14.0% had a conversation with only their PCP. Additionally, 11.8% reported they weren’t sure with whom they had an ACP conversation. At the time of the survey, a large majority had completed or revised their existing advance directive (78.5%), about half had a Portable Order for Life Saving Treatment medical order signed by their provider (49.5%), and the majority (88.2%) had appointed a Health Care Representative (HCR). In the survey, we defined an HCR as “a person who is going to make healthcare decisions for me if I cannot speak for myself.”

**Figure 1.**
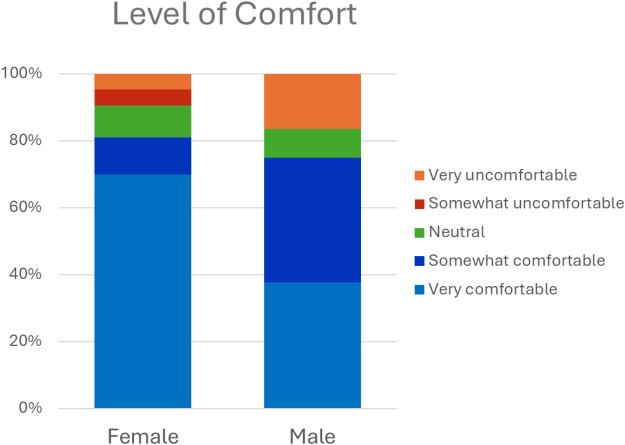
Level of comfort with having conversations about advance care planning with healthcare professionals in primary care practice by gender. Figure 1 long description.

We asked respondents to rate their comfort level in having ACP conversations with healthcare professionals in primary care practice. Sixty percent reported being very comfortable, 19% were somewhat comfortable, 9% were neutral, and 12% were somewhat or very uncomfortable. When stratifying comfort level by gender, we observed that 70% of females and 38% of males rated themselves as very comfortable (see Fig. 1). Additionally, 5% of females and 17% of males reported being very uncomfortable, an exploratory, but statistically significant difference in comfort level by gender (*p* = 0.015).

**Figure 2.**
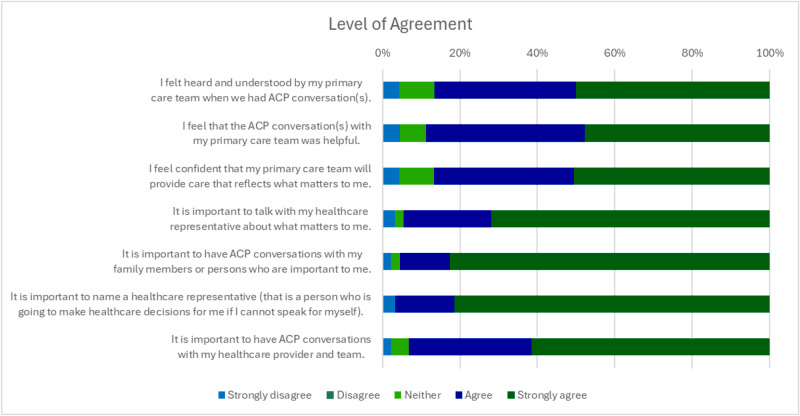
Level of agreement about experiences of having ACP conversations with a healthcare team. Figure 2 long description.

**Figure 3.**
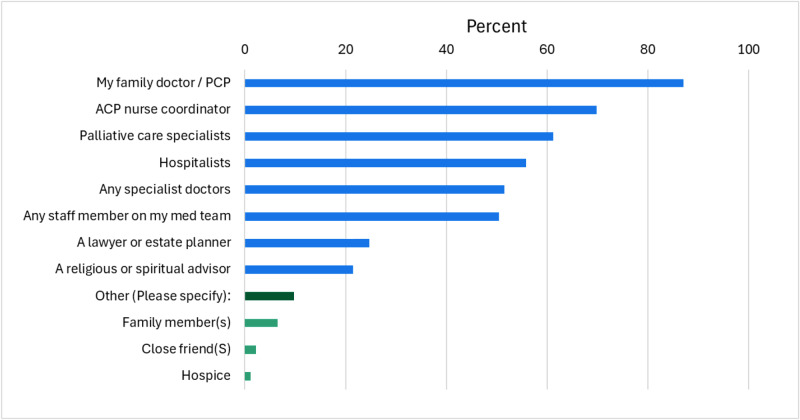
Preferred persons for advance care planning discussions (participants could choose multiple responses). Figure 3 long description.

Among respondents, 93% thought ACP discussions with their PCT were important, and 89% found them helpful. Nearly all (96%) thought it was important to have these conversations with family or to talk with HCR about what matters most to them (95%). Eighty-seven percent felt heard and understood, and confident their PCT would honor their preferences, and 97% agreed that naming an HCR was important (see Fig. 2). When asked who should be involved in ACP discussions, patients most frequently selected their PCP (87%), ACP-NC (70%), and palliative care specialist (61%) (see Fig. 3).

### Qualitative interviews with patients

Semi-structured interviews with patients and caregivers allowed us to better understand their experiences with the ACP conversations. Participants discussed feeling comfortable being referred to the ACP-NC for additional support, appreciating the time spent with ACP-NC, and valuing her motivation and accountability in helping complete the related paperwork. For demographics of the interviewed qualitative participants, see Table 2.

**Table 2.** Demographics of interviewed patients (*n* = 10) Table 2 long description.

	Number of patients
Sex	
Male	5
Female	5
Health status	
Stable	2
Moderately stable	1
Moderately unstable	1
Unstable	1
Moderately ill	2
Very ill	1
Deceased^a^	2
Age	
70–75	2
76–80	3
81–86	3
Missing^b^	2

a Caregivers participated in interviews for deceased patients; all other participants were the patients.

b The deceased patient's age at death was missing.

## Participants were generally happy being referred to ACP-NC for additional support

Participants stated feeling comfortable being referred from their clinician to the ACP-NC for follow-up and further ACP conversations. The referral process was facilitated by a warm handoff from their PCP, which participants identified as an important factor in fostering trust and continuity. This approach allowed the referral to the ACP-NC to be perceived as a natural extension of the existing patient-clinician relationship. One participant described the deep trust she had with her PCP as the foundation of her participation with the ACP-NC, stating:

I would say, because of the way Dr. <Name> has been, I just trust her, and probably anyone she refers me to, I trust, because I trust her. -Participant 7

This perspective was held among the majority of interviewees, however, 1 participant indicated they preferred having the conversation with their PCP and did not do much work with the ACP-NC.

I think it’s nice [the ACP conversation] comes from the family physician— the one the person is comfortable with. Another person they don’t know coming in, yeah, I think I’d be rather nervous about that. -Participant 1

For this participant, the benefit of extending the patient-clinician relationship to include the ACP-NC was challenging due to the exclusive trust they experienced with their PCP.

## Participants appreciated the time the ACP-NC spent allowing deeper conversations

Participants appreciated the time the ACP-NC spent with them discussing their specific situation, desires, wants, and needs. They appreciated her deep listening and thoughtful responses in a relaxed environment, which was reported as a contrast to the limited available time from PCPs. The time the ACP-NC was able to spend allowed for detailed assistance with end-of-life logistics and served as a sounding board for families. Their needs were addressed with care and attentiveness for sensitive discussions. Additionally, the time allowed her to offer practical guidance to caregivers who often accompanied patients in these conversations, such as communication strategies and conversational scripts to facilitate discussions with patients.

She’s asking questions to me, and then I’m asking questions to her. It’s like she’s taking some time to spend some real time with me over the phone and is very calm. -Participant 3

I think her taking the time—I think we had an appointment that was just about this where she took a whole hour just dealing with this [ACP]. The advance directive, if nothing else, made us have to know—made me have to know what he wanted. Made it more important. It was extremely valuable. -Participant 9

By intentionally providing substantial dedicated time to the ACP topic, patients felt appreciated and heard.

## Participants reported that the ACP-NC provided motivation and accountability

The ACP-NC maintained consistent follow-up with patients to ensure full reflective conversations and completion of necessary documentation, which led to her serving as a motivator and accountability partner. Patient participants expressed appreciation for the ACP-NC’s regular follow-ups and reminders, doing so in a manner that was supportive rather than intrusive, which led to valuable opportunities for information sharing, clarification, and support. Several patient participants described contemplating ACP and the related documentation, but had put it off or had not found the time. One said:

Well, to be quite honest, I’d been thinking about [ACP] for a while ever since my parents’ death. Especially after watching my sister go through it. I just never got around to it. When we had the meeting with [ACP-NC], that really put it in motion. It got it done. -Participant 8

This patient participant, like others, found that the ACP-NC served as a catalyst for ongoing ACP conversations and helped to complete the necessary paperwork.

What we didn’t understand, they helped. They were always willing to help. Without the two of them [ACP Nurse and PCP], it [ACP documentation] would never have gotten done. To look back now, how much we needed it at one point. Neither one of us thought he was gonna die when that was happening. -Participant 9

By having the ACP-NC as a motivator and accountability partner, this caregiver participant was able to proactively prioritize these conversations and get the related documentation completed prior to the loss of their loved one.

### Qualitative interviews with clinicians

Six clinicians were interviewed (including 4 physicians, 1 physician associate, and the ACP-NC). Four of them received the ACP training, and all 6 took part in ACP conversations and referrals. The physicians and a physician associate all worked with and used the ACP-NC as a resource to facilitate ACP conversations.

## Perceived benefits of a dedicated ACP-NC role: Enhanced efficiency, focused communication, and improved quality of care

All participants cited time as the single biggest barrier to having in-depth ACP conversations and felt the ACP-NC provided a time-saving advantage. ACP-NC’s flexibility in having these conversations around patients’ schedules was appreciated. Participants described that even when they set aside dedicated time to have ACP conversations, other urgent medical priorities crowded out this topic, whereas conversations with the ACP-NC were more likely to stick to that topic, which focused solely on the conversations. A general perception from clinician participants directly related to this work was that the ACP-NC allowed the clinic to offer higher-quality care:

I think that having somebody [ACP-NC] take a piece of that [ACP] burden lifts the weight of the conversation from my shoulders. Also the reminder of the importance of the conversation is a big piece of what she does. I see that not having her would add to the stress and the already overworked nature of what we do. It would also detract from the quality of care that we’re able to provide, not only in our clinic, but more in the hospital setting. Not being able to have documented, clear conversations with the inpatient side of things is gonna lead to worse outcomes in a system that’s already struggling to provide care to patients. If we have this [ACP] that’s already done ahead of time, it’s gonna take that burden off of an expensive, time-consuming system that we already have. It’s giving us a little bit of a reprieve from the weightiness of medicine, but also easing the burden of patients and the system. -Clinician 6

The ACP-NC role was seen as an indispensable and valued time-saving and focus-creating tool that provided a meaningful contribution to patients and physicians alike, in its ability to improve outcomes and care.

## Clinicians viewed their role as part of a team along with the ACP-NC in delivering ACP conversations

The participants described their role as being collaborative as a team member, which allowed for time and opportunity to learn about many more patients and their healthcare priorities.

It really should be a collaborative process. I hope that everybody sees it this way, but it really shouldn’t fall just onto one person. I think that having different people looking at this topic from different viewpoints is really helpful because we each have our own biases when we’re looking at patients. Having a nurse, a PA, a physician, a specialist, different people thinking about the topic, it really puts the responsibility onto all of us. We maybe can identify more patients that need the conversation than if just one of us alone was doing it. I really do think it’s a collaborative effort; that we all really should be doing it. -Clinician 6

Clinicians felt a shared responsibility with the ACP-NC and were grateful for her supportive partnership. Additionally, the 2 way communication process raised awareness and fostered motivation for clinicians to learn, grow, and do this much-needed, but challenging work with patients.

The most helpful thing has been just knowing that <ACP-NC> is there and being able to refer to her, and also it has been very helpful that she sends me feedback. Because you never know how if you’re sending the right—as a family doc, I get these sanitized reports back from consultants, and I have to read between the lines to decide like, “Was that an appropriate consultation? Are they annoyed, or are they—did they feel like this was a good use of their time?” <ACP-NC>’s. consistent feedback that “I think this was helpful. Thank you. This is what I did. I arranged.” The two-way conversation is incredible, and that has been motivating for me to continue doing what I’m doing. -Clinician 2

Having a dedicated staff member in this role highlighted the importance of team-based care, with continuous quality improvement in clinic, making this process more feasible.

## Discussion

Having an ACP-NC in the PCT, who has dedicated time to engage in ACP conversations with patients, was well accepted and likely increased the frequency of ACP. The availability of the ACP-NC as a resource and/or providing SIC training to clinicians improved the clinicians’ awareness and willingness to engage in ACP discussions. Despite the common belief that patients do not want to talk about ACP (Malhotra and Chaudhry 2023), more than two-thirds of patients in our sample reported feeling comfortable having these conversations. Notably, though, discomfort levels differed by gender: only 5% of females reported being very uncomfortable, compared with 17% of male participants. The majority of patient participants of both genders recognized the importance of ACP and preferred to discuss it with their PCPs and/or PCT members, including the ACP-NC.

In the interviews, patients expressed appreciation for the ACP-NC, who initiated the ACP conversation, spent time listening, followed up, and assisted them in thinking through their values, goals, and preferences. The emphasis on the ACP-NC’s role in starting the discussion aligns with prior research showing that patients believe it is the healthcare professionals’ role to initiate ACP conversations (Bernard et al. 2020). This suggests an opportunity for early initiation of ACP conversations in primary care settings. Clinicians in the clinic saw the ACP-NC as a valuable resource to deliver high-quality ACP when they did not have enough time to do so by themselves.

Creating an ACP-NC position with designated time seems to be an acceptable and effective approach to address the latent needs among patients. Although we did not evaluate the long-term impact of the early ACP conversations beyond the Family Medicine clinic, literature suggests that early ACP conversations contribute to the delivery of more patient-centered, goal-congruent care and reduce the decision burden felt by family members. (Bischoff et al. 2013; Dalmau-Bueno et al. 2021; Degenholtz et al. 2004; Detering et al. 2010; Wright et al. 2008).

Having an ACP-NC has other advantages, particularly for patients who are not comfortable talking about ACP. For patients and families who are not immediately ready to open up to ACP conversations, it allows them to warm up to the topic, as the ACP-NC was able to spend considerable time with them (Miller et al. 2019). The role of ACP-NC facilitating the ACP conversation is within the scope of nursing practice (Resendes 2024). Therefore, PCPs can save time by having a warm handoff of the ACP conversation to the nurses on their team.

Despite these advantages of having ACP-NCs, creating and securing the ACP-NC position in primary care settings is challenging. In the current healthcare system, primary care clinics tend to be underfunded, and financial resources to secure the ACP-NC position are limited. Some primary care clinics may not have nurses on their teams. Furthermore, in most primary care clinics, ACP conversations conducted by non-billing clinicians, such as registered nurses, do not generate their own revenue (Department of Health and Human Services 2015). However, nurses, as compassionate and unique advocates of patients, may be better positioned to hold these conversations. Our grant funded the ACP-NC position, along with additional resources to educate and evaluate the ACP conversation processes. Although the grant sustained this work for several years, the position was discontinued once the funding ended.

Our study has limitations. Our survey response rate was 60% and moderate, but there is a possibility of respondents’ bias, as we do not know the reasons for the non-responses, such as advancing illness. There was also a time lag between the last ACP conversation and the time of the interviews, resulting in potential recall bias. The number of interview participants was small for patients and clinician groups. This study was conducted in 1 primary care clinic in an academic setting; therefore, the generalizability of the findings is limited. A large proportion of participants in this study were highly educated and white. Different findings may emerge in clinical settings that are more racially, socioeconomically, or educationally diverse. Future research could involve pragmatic trials across multiple primary care settings to evaluate the effectiveness of this approach. Additionally, a cost analysis of the ACP-NC position would be an important avenue for further investigation.

Most patients in our study were open to engaging in ACP discussions, presenting an important opportunity for providers to initiate these conversations. Integrating an ACP-NC within PCTs may represent an acceptable and effective approach to promote the early initiation of ACP in primary care settings.

## Supporting information

10.1017/S1478951526102508.sm001 Devarajan et al. supplementary material 1 Devarajan et al. supplementary material

10.1017/S1478951526102508.sm002 Devarajan et al. supplementary material 2 Devarajan et al. supplementary material

10.1017/S1478951526102508.sm003 Devarajan et al. supplementary material 3 Devarajan et al. supplementary material

## Supplementary material

The supplementary material for this article can be found at https://doi.org/10.1017/S1478951526102508.

## Table 1 Long description

The table presents demographic data of 93 survey participants, focusing on age, gender, ethnicity, race, education, and survey type. The average age is 79.9 years, with a range from 52 to 95 years. Females constitute 68.8% of the participants, while 92.5% identify as White. Most participants have completed graduate school or higher (35.9%). The survey was conducted via email (60.2%) and paper/phone (39.8%). The response rate was slightly higher for email surveys (60.2%) compared to paper/phone (58.7%). The data suggests a predominantly older, educated, and White demographic, with a preference for email surveys.

Navigate back to Table 1.

## Figure 1 Long description

A stacked bar chart titled 'Level of Comfort' compares the comfort levels of female and male respondents regarding Advance Care Planning conversations. The chart includes five categories: very uncomfortable, somewhat uncomfortable, neutral, somewhat comfortable and very comfortable. Each category is represented by a different color. The female bar shows a larger proportion of respondents feeling very comfortable compared to the male bar. Conversely, the male bar has a higher proportion of respondents feeling very uncomfortable compared to the female bar.

Navigate back to Figure 1.

## Figure 2 Long description

The graph shows the level of agreement on various aspects of ACP conversations. The x-axis represents the percentage from 0 to 100 percent, while the y-axis lists statements about ACP conversations. The statements include feeling heard by the primary care team, finding ACP conversations helpful, confidence in care reflecting personal values, importance of conversations with healthcare representatives and family, naming a healthcare representative and having ACP conversations with the healthcare provider and team. Each statement is represented by a horizontal bar divided into sections indicating levels of agreement: strongly disagree, disagree, neither, agree and strongly agree.

Navigate back to Figure 2.

## Figure 3 Long description

A bar graph illustrating preferred persons for advanced care planning discussions. The x-axis is labeled 'Percent' ranging from 0 to 100. The y-axis lists various roles: 'My family doctor / PCP' at 87 percent, 'ACP nurse coordinator' at 70 percent, 'Palliative care specialists' at 61 percent, 'Hospitalists' at 50 percent, 'Any specialist doctors' at 48 percent, 'Any staff member on my med team' at 40 percent, 'A lawyer or estate planner' at 30 percent, 'A religious or spiritual advisor' at 20 percent, 'Other (Please specify)' at 10 percent, 'Family member(s)' at 5 percent, 'Close friend(s)' at 3 percent and 'Hospice' at 1 percent. Each bar represents the percentage of respondents who selected each category.

Navigate back to Figure 3.

## Table 2 Long description

The table presents demographic data for 10 interviewed patients, split evenly between males and females. Health status varies, with two patients stable, one moderately stable, one moderately unstable, one unstable, two moderately ill, one very ill, and two deceased. Age distribution includes two patients aged 70-75, three aged 76-80, three aged 81-86, and two with missing age data. The data highlights a diverse range of health conditions and age groups among the patients, with a note that age at death is not available for deceased patients.

Navigate back to Table 2.
