# Supplementary material for: Impact of nurse-led advance care planning in a primary care setting
Source: Palliat Support Care. 2026 May 12;24:e140. doi: 10.1017/S1478951526102508 (PMC13202411; doi:10.1017/S1478951526102508)
Supplement: Devarajan et al. supplementary material 1 — Devarajan et al. supplementary material [file S1478951526102508sup001.docx]

**Advanced Care Planning Project in Family Medicine**

**Patient survey:**

Thank you for participating in this research study of Advance Care Planning (ACP). You were asked to participate in this survey because you had conversations with the ACP nurse coordinator (Erin Gallivan, RN) and/or your primary care provider about your goals, values, and preference for future medical care.

In the survey below, we will name the conversation you had an “ACP conversation”, which is intended to explore your priorities, important goals, and values in your life which may affect your future medical care, in case you get very sick and become incapable of speaking for yourself. The survey should take about 5-10 minutes.

1. Who did you have an ACP conversation with?

☐ The ACP nurse coordinator only (Erin Gallivan, RN)

☐ My primary care provider only

☐ Both the ACP nurse coordinator and my primary care provider

☐ I am not sure

1. The next set of questions asks about your Advance Care Planning conversation(s) (i.e. conversations about your goals, values, priority and preferences).

Please choose your level of agreement with the following statements:

|  | Strongly disagree | Disagree | Neither | Agree | Strongly agree |
| --- | --- | --- | --- | --- | --- |
| 1. It is important to have ACP conversations with my healthcare provider and team. | 1 | 2 | 3 | 4 | 5 |
| 1. It is important to have ACP conversations with my family members or persons who are important to me. | 1 | 2 | 3 | 4 | 5 |
| 1. It is important to name a healthcare representative (i.e. a person who is going to make healthcare decisions for me if I cannot speak for myself). | 1 | 2 | 3 | 4 | 5 |
| 1. It is important to talk with my healthcare representative about what matters to me. | 1 | 2 | 3 | 4 | 5 |

1. Next, we would like to ask you about your experience of having Advance Care Planning conversation(s) with your healthcare team.

Please choose your level of agreement with the following statements:

|  | Strongly disagree | Disagree | Neither | Agree | Strongly agree |
| --- | --- | --- | --- | --- | --- |
| 1. I feel that the Advance Care Planning conversation(s) with my primary care team was helpful. | 1 | 2 | 3 | 4 | 5 |
| 1. I felt heard and understood by my primary care team when we had ACP conversation(s). | 1 | 2 | 3 | 4 | 5 |
| 1. I feel confident that my primary care team will provide care that reflects what matters to me. | 1 | 2 | 3 | 4 | 5 |

1. Please choose your answer to the following questions:

| 1. Have you completed an Advance Directive? | Yes | No | Not sure |
| --- | --- | --- | --- |
| 1. Do you have a Portable Order for Life Sustaining Treatment (POLST) signed by your provider? | Yes | No | Not sure |
| 1. Have you appointed a Healthcare Representative (that is a person who is going to make healthcare decisions for me if I cannot speak for myself)? | Yes | No | Not sure |

1. Please rate your level of comfort with having conversations about Advance Care Planning with healthcare professionals in primary care practice.
   - Very uncomfortable
   - Somewhat uncomfortable
   - Neutral
   - Somewhat comfortable
   - Very comfortable
2. Which professional or professional groups would you want to be involved in discussing Advanced Care Planning topics? (**Select all that apply**)

- My family doctor/primary care provider
- ACP nurse coordinator
- Any staff member who is part of my medical team, for example, nurses, medical assistants, social workers, or mental health experts
- Any specialist doctors (if I have them)
- Palliative care specialists (Team of professionals including doctors, social workers, and nurses, who work with patients and their loved ones who have very serious medical conditions.)
- Hospitalists (Doctors who takes care of you when you are admitted to the hospital)
- A religious or spiritual advisor
- A lawyer or estate planner
- Other (Please specify): _______________

1. Is there anything you think your care team; your provider, office staff, and the ACP nurse coordinator could have done better during the Advance Care Planning conversation? Please comment below if you have any suggestions.

1. What is your age? __________
2. What is your gender?

- Female
- Male
- Non-binary
- Prefer not to answer

1. Are you of Hispanic or Latino origin or descent?

- No, not Hispanic or Latino
- Yes, Hispanic or Latino
- Prefer not to answer

1. What is your race? (Select all that apply)

- American Indian or Alaska Native
- Asian
- Black or African American
- Middle Eastern and Northern African
- Native Hawaiian or Pacific Islander
- White
- Prefer not to answer

1. What is the highest level of education you have completed?

- Some elementary or middle school
- Some high school, but no diploma
- High school diploma or GED
- Some college, no degree
- 2-year or 4-year college degree
- Graduate school or higher
- Other, please specify _______________

1. What language(s) do you mainly speak at home?

***You’ve reached the end of the survey. Thank you for your participation.***
