# Supplementary material for: Impact of nurse-led advance care planning in a primary care setting
Source: Palliat Support Care. 2026 May 12;24:e140. doi: 10.1017/S1478951526102508 (PMC13202411; doi:10.1017/S1478951526102508)
Supplement: Devarajan et al. supplementary material 2 — Devarajan et al. supplementary material [file S1478951526102508sup002.docx]

**Patient Interview Guide**

**Advanced Care Planning**

The questions below are the general topic areas we will explore with interview participants. These questions will be modified in light of what is learned during the study and to fit the expertise of the interviewee*.*

*Thank you for participating in this interview. We are speaking with you today because we’d like to learn more about your experiences with Advanced Care Planning conversations you’ve had with [insert names of providers] at [name of practice]. We want to understand the steps involved in the process and what could be done to improve these conversations for other patients. We really value your perspective, and we’re interested in your candid thoughts, so please do not hesitate to share your opinions.*

*Introduce yourself and second interviewer if applicable.*

*Did you have a chance to review the information sheet? Ensure participant has a copy. Do you have questions?*

*Describe how the interview audio transcripts are de-identified and handled: Audio recordings will be professionally transcribed, and any information in the interview that could be used to identify you will be stripped from the transcripts. These transcripts will only be seen by and shared with the research team.*

Start recording: *Do I have your permission to record this interview?*

1. **I’d like to know a bit more about you. Can you tell me about yourself?**

*Now, I’d like to understand a bit more about your health and where you go for health care.*

1. **Tell me about where you go for health care.**

- How long have you been going to [name of practice]?
- How long have you been seeing [PCP]?
- Who do you usually see when you go to [name of practice]?

1. **When you think back over the last year, what’s been going on with your health?**
   - Why do you go to [name of practice]?
   - How often do you go?
   - What’s been going on with your health?
   - What have your health needs been?

*We understand that you’ve had an advance care planning conversation with [insert name of provider(s) at practice] approximately around [insert timing if available]. We’d like to hear about your experience with that.*

1. **To start, can you walk me through what that experience was like?**

- Who approached you about advanced care planning?
- When were you approached?
- How was the conversation brought up?
- Can you walk me through all the steps?
- Describe the process for me.

1. **What was it like to have this conversation with [ACP-NC (if applicable)]?**

- What were the types of things you discussed?
- What questions did you have?
- What, if anything, was helpful about this conversation? Why?
- What did you need from your family to complete the process?
- What, if anything, did you need from your doctor?
  - From other clinicians/team members at your practice?

1. **What was it like to have this conversation with [PCP (if applicable)]?**

- What were the types of things you discussed?
- What questions did you have?
- Where did you need more information before you could make a decision?
- What, if anything, did you need from your family to complete the process?
- If applicable, how did this conversation compare to the conversation with [ACP-C]?

1. **I understand there were also some forms you had to fill out. Can you tell me about your experience completing the forms?** [Patient may have received an Advanced Care Directive Booklet blue last section, the Oregon Online form, a pink POLST form. Use those descriptors if patient needs help remembering.]

- What questions did you have?
- Who helped you fill them out?
- What did you need your family’s help with?
- What did you need your practice’s help with?

1. **What was your family’s role in this process?**
2. **When you think about your advance care planning conversation(s), what was uncomfortable about the process?**

- What would have made it more comfortable?

1. **When you think about your advance care planning conversation(s), what was comfortable about the process?**

- What did [ACP-NC] do to make you feel comfortable?
- What did [PCP] do to make you feel comfortable?

1. **Who else would you have liked to talk to about advance care planning?**

- Why?

1. **How would you feel about having an advance care planning conversation with someone else [not ACP-NC or PCP] who works at clinic?**
2. **What do you think would make advanced care planning conversations better for other patients?**
3. **What else would you like to share about advanced care planning that we haven’t discussed?**

*Thanks so much for taking the time to speak with us today!*
