# Supplementary material for: Impact of nurse-led advance care planning in a primary care setting
Source: Palliat Support Care. 2026 May 12;24:e140. doi: 10.1017/S1478951526102508 (PMC13202411; doi:10.1017/S1478951526102508)
Supplement: Devarajan et al. supplementary material 3 — Devarajan et al. supplementary material [file S1478951526102508sup003.docx]

**Clinician Interview Guide**

**Advanced Care Planning**

*Thank you for participating in this interview. We are speaking with you today because we’d like to learn more about your experiences with Advanced Care Planning (ACP) at your practice. We’d like to better understand how you see your role in advanced care planning with patients, your experience with the training, and the potential role of other team members in serious illness conversations.*

*Did you have a chance to review the information sheet?* Ensure participant has a copy. *Do you have questions?*

Describe how the interview audio transcripts are de-identified and handled: *Audio recordings will be professionally transcribed, and any information in the interview that could be used to identify you will be stripped from the transcripts. These transcripts will only be seen by and shared with the research team.*

Start recording: *Do I have your permission to record this interview?*

**1. First, please tell me about yourself.**

- What is your background?
- What is your role here?
- How long have you worked here?

*To start, I’d like to learn more about the ACP/serious illness conversation training that you received.*

**2. Please tell me about your experience with the serious illness conversation training.**

- What did you learn?
- What was the training like?
- What, if anything was new to you?
  1. **How prepared were you to have advance care planning conversations with your patients following the training?**
- In what areas do you need more training?
- What would help you feel more prepared?
- What has helped you feel prepared?
- What, if anything, have you done differently when having ACP conversations with your patients?

*I’d like to better understand your experience with ACP discussions now.*

**4. What is your role in ACP conversations?**

**5. Can you help me understand your workflow for having ACP conversations?**

- How do you know when it’s time to have this discussion with a patient?
- What prompts you to have this discussion?
- Who’s involved in the process?
- What information, if any, do you collect prior?
- What are the steps? What happens next**?**

**6. Please tell me about your experience with ACP conversations with your patients?**

- How do you initiate ACP conversations?
- What aspects of these conversations, if any, are difficult to complete?
- What barriers, if any, do you experience?
- What is helpful for having these conversations?
- How would you describe your comfort discussing these topics?
  - What aspects are comfortable? Why?
  - What aspects are uncomfortable? Why?

**7. Can you tell me about the documentation requirements that go along with ACP?**

- What are you required to do following a conversation?
- What, if any, aspects are burdensome?
- What, if anything, could be completed by another member of your team?

**8. From your perspective, who do you think should have these conversations with patients?**

- Why?
- What about [role] makes them a good option for this responsibility?

**9. Are there any aspects of ACP conversations that could be completed by another care team member?**

- Why or why not?
- Please describe the specific component someone else could complete.
- Is this something that could be completed by someone at OHSU/but not at the practice? Why?
- Which aspects are important for a clinician to do? Why?
- What other roles/members of your team could facilitate these conversations?
- What about [role] makes them a good option for this responsibility?

**10. What else do you think is needed to improve how ACP conversations are conducted in your practice?**

**11. What else would you like to share that would be important for understanding ACP conversations?**

**12. What questions do you have for me?**

Thanks so much for taking the time to speak with us today!
